# The modifications of enclave mentality and identity issues in a post-conflict society: a critical review of a psychopolitical analysis of Cyprus

**DOI:** 10.3402/ejpt.v6.27328

**Published:** 2015-02-10

**Authors:** Bülent Evre

**Affiliations:** Department of Political Science, Near East University, Lefkoşa, Turkey. bulentevre@yahoo.com

Review of *Enemies on the couch: A psychopolitical journey of war and peace*, by Vamık D. Volkan. Durham: Pitchstone Publishing, 2013. 504 pp. ISBN 978-1939578037. $22.99 Hardback.

Exploring societies in conflict or postconflict, which is relevant to conflict resolution, conflict management, peacebuilding, and peacekeeping initiatives requires an interdisciplinary research. Vamık Volkan who is considered to be a prominent expert in the psychology of war-torn societies works in an interdisciplinary framework of political psychology. Volkan's recent book entitled *Enemies on the couch* offers his recent observations and evaluations of societies both in conflict and postconflict. Although each chapter of the book deserves a distinct appraisal, this review is exclusively of the chapter regarding Cyprus entitled “A Return to Cyprus: The Presence of Invisible Walls.”

Volkan aims to illustrate in general that the basic aspects of individuals and large-group psychology in societies after major trauma remain immutable, and in particular this is valid for Cyprus as well. Within this context, he argues that the Cypriot Turks’ experience of living in enclaves in pre-1974 circumstances still continue to affect their large-group identity, and that other external factors have led to more identity confusion. However, Volkan's observations and evaluations on Cyprus seem to suffer from conceptual ambiguity, lack of evidence, and logical flaws.

The scope of the study on Cyprus is limited to the Cypriot Turks, particularly those living in post-1974 era, which is characterized as a society in postconflict. Volkan begins his analysis of the Cypriot Turks on the basis of societal trauma, and distinguishes between the concepts of “concrete enclave” and “invisible enclave,” which are central ideas to his main argument. Accordingly, the concept of concrete enclave is defined by pre-1974 circumstances in which the Cypriot Turks were forced by the Cypriot Greeks to live in subhuman conditions in enclaves, geographically confined to 3% of Cyprus for 11 years. While the concept of invisible enclave refers to post-1974 conditions in which even though the Cypriot Turks declared independence and established a separate state in Northern Cyprus, they continued to live in isolation from the world community because their state was not recognized by any country except for Turkey.

Although there are dramatic changes between the pre- and the post-1974 conditions in Cyprus, Volkan's distinction between concrete and invisible enclave is questionable. His choice of the word “invisible” is not obvious enough, because it may have several meanings depending on the context in which it has been employed. If it refers to post-1974 circumstances, what makes them invisible? If they are invisible indeed, how come Volkan has been able to discover such conditions? However, as he himself admits, for his study was “not a strictly scientific one, meaning [he] did not receive answers to specific questions and then measure them according to any designated methodology” (p. 365), it is not possible to find out through which methodology he has identified “invisible” enclave.

On the contrary, if he insists on the existence of enclave even in post-1974 circumstances, then it can be accepted as a matter of size of the enclave. Although the size of the enclave was relatively small in the pre-1974 era, it has become relatively larger in the post-1974 era. Thus, the distinction between small and large enclaves would be more accurate to refer to the modifications of what he calls “enclave mentality” on the island.

In addition to his distinction between the concrete enclave and the invisible one, Volkan has developed the concept of “enclave mentality” through his 2007 and 2008 study in which data are obtained from the interviews with older individuals and his daily activities. He claims that “enclave mentality continues to be present even after the traumatic environment no longer exists” among the Cypriot Turks (p. 367). Volkan suggests that even though the impact of massive social trauma is repressed or denied, it still manifests itself in multiple ways in new generations. Nevertheless, he argues that the younger generation born to parents with what he calls “enclave mentality” is not aware of this.

However, the author provides no evidence to support this claim. He does not illustrate how massive trauma manifests itself in new generations. If the impact of massive trauma continues to be present, why are the new generations not aware of this, and how has he found it? Answers to these questions are not found in the study. Therefore, Volkan's claim remains just a hypothesis yet to be tested.

On the contrary, for Volkan, some international actors’ efforts to construct a Cypriot “ethnic group” or “nation” within the context of finding a resolution to the Cyprus conflict is another external factor, which has modified and increased what he calls “identity confusion.”

The author puts forward the debates over national identity in Turkey, what he describes as “the process of changing Turkishness” as another major external factor which “disrupted the large-group identity investments of Cypriot Turks” (p. 372). Through his interviews with dozens of young and old persons, he has found that there is no one primary large-group identity of Cypriot Turks, but different identities such as a Turk, a secular Turk as Kemal Atatürk, a religious Turk, a Cypriot who is like a Greek Cypriot, a Cypriot different from a Greek Cypriot, a European, and so on. And he concludes with his belief that revealing peoples’ emotions both in Northern Cyprus and Turkey has played a crucial role in “taming exaggerated emotions associated with identity confusion” (p. 374).

Nevertheless, there are several questionable points here. First, his diagnosis of “identity confusion” can be critically appraised in terms of relevant literature in general and Eriksonian theory in particular. Although there is a growing literature on identity which emphasizes that individuals may hold multiple identities or roles (for some of them, see Deaux 1996; Laing 1988; Lickel et al. 2000; Sieber 1974), Volkan disregards the relevant literature and accordingly multiplicity of identities.

Furthermore, the term “identity confusion” seems to have been borrowed from Eriksonian developmental theory. Erikson describes “identity versus role confusion” as the fifth stage of psychosocial development in which adolescents are “primarily concerned with what they appear to be in the eyes of others” and develop a new sense of identity. Yet, there is a danger of role confusion. They may not be able to settle on occupational, sexual, or ideological identity. In other words, there may appear “complete loss of identity” (Erikson 1987). Within this framework, Volkan's finding of young generations’ identification with different identities does not conform to identity confusion either. If they had no answer about who they are, it would be true to say that they are in a state of identity confusion. Conversely, the finding displays that they have identified with at least one social identity. Not holding one primary large-group identity cannot be evaluated as identity confusion, but at best as multiplicity of identity.

The other arguable aspect is his assertion that some international actors have made some efforts to create a Cypriot “ethnic group” or “nation” in the attainment of a solution to the Cyprus conflict. It is noteworthy to underline that Cyprus peace talks have been carried out under the auspices of the United Nations in an internationally recognized manner, and so far no resolution proposal consisting of a Cypriot nation or ethnic group has been put on the negotiating table (for some assessments of Cyprus negotiations, see Bayülken 2001; Claire 2006; Polyviou 1980). The term “Cypriot” in all settlement proposals has been regarded as neither a nation nor an ethnic group but as a citizenship on the basis of territory. For instance, the latest comprehensive settlement proposal, which is commonly known as Annan Plan, envisages Cypriot citizenship as a postnational identity comprising both Turkish Cypriot and Greek Cypriot citizenship, which would be complementary status.^1^

Moreover, the author's claim that the debates on national identity in Turkey as another major external factor “disrupted the large-group identity investments of Cypriot Turks” is open to question too. First of all, it is to be taken into consideration that the recent national identity issues in Turkey have been particularly debated around Turk, Turkeyian (*Türkiyeli* in Turkish), and Kurd identities. That is to say that the most controversial debate in Turkey has been between Turkish and Kurdish identities, and in Northern Cyprus, it has been between Cypriot and Turkeyian identities. Thus, there is a problem of oversimplification inasmuch as there is no direct relationship between the recent national identity debates in Turkey and the identity issues among the Cypriot Turks.

Consequently, despite these criticisms, Volkan's work offers remarkable insights into the concept of enclave mentality, which refers to the psychology of individuals regarding their experience of living in enclaves, and its relation to large-group identity within the context of Cyprus. However, there exist conceptual ambiguity, lack of evidence, and logical flaws in the construction of his argument.

*Bülent Evre* Department of Political Science Near East University, Lefkoşa, Turkey bulentevre@yahoo.com

## Footnotes

1 Article 3(2) of the Annan Plan states that “All Cypriot citizens shall also enjoy internal constituent state citizenship status. Like the citizenship status of the European Union, this status shall complement and not replace Cypriot citizenship.”
